# Potential Active Constituents from *Opophytum forsskalii* (Hochst. ex Boiss.) N.E.Br against Experimental Gastric Lesions in Rats

**DOI:** 10.3390/ph15091089

**Published:** 2022-08-31

**Authors:** Ahmed I. Foudah, Fawwaz Khalaf Aloneizi, Mohammad H. Alqarni, Aftab Alam, Mohammad Ayman Salkini, Hamad M. Abubaker, Hasan S. Yusufoglu

**Affiliations:** 1 Department of Pharmacognosy, College of Pharmacy, Prince Sattam Bin Abdulaziz University, Al Kharj 11942, Saudi Arabia; 2 College of Health Sciences, Al-Rayan Colleges, Madinah Al Munawwarah 42541, Saudi Arabia; 3 Department of Pharmacognosy & Pharmaceutical Chemistry, College of Dentistry & Pharmacy, Buraydah Private College, Buraydah 81418, Saudi Arabia

**Keywords:** Saudi Arabia, *Opophytum forsskalii*, indomethacin, stress, necrotizing agents, gastro-protective

## Abstract

*Opophytum forsskalii* (*O. forsskalii*) is a desert plant that belongs to the Aizoaceae family. Although it is a natural food source for Bedouin tribes in northern Saudi Arabia, there is little information on its active metabolites. Therefore, the secondary metabolites of the hydroalcoholic extract from the leaves of this species were analyzed by liquid chromatography-mass chromatography (LC-MS). LC-MS identified a total of 30 secondary metabolites. These compounds represented two main categories among sixteen classes. Among them, flavonoids represented the largest proportion with eleven metabolites while fatty acids provided seven compounds. In addition, the extract was evaluated for its gastroprotective effect against gastric lesions induced by different models, such as indomethacin, stress, and necrotizing agents (80% ethanol, 0.2 mol/L NaOH, and 25% NaCl), in rats. For each method, group 1 was used as the control group while groups 2 and 3 received the leaf extract at doses of 200 and 400 mg/kg, respectively. The ulcer index (UI) and intraluminal bleeding score (IBS) were measured for each method. In addition, gastric tissue from the ethanol method was used for the analysis of nonprotein sulfhydrates (NP-SH), malondialdehyde (MDA), total protein (TP), and histopathologic evaluation. Pretreatment with *O. forsskalii* significantly decreased UI (*p* < 0.01) and IBS (*p* < 0.01) at 400 mg/kg. Pretreatment with *O. forsskalii* significantly improved total protein levels (*p* < 0.01) and NP-SH (*p* < 0.001) compared to the ethanol ulcer groups. MDA levels increased from 0.5 to 5.8 nmol/g in the normal groups compared to the ethanol groups and decreased to 2.34 nmol/g in the *O. forsskalii pretreatment*. In addition to the gastroprotective markers, histopathological examination of gastric tissue confirmed the gastroprotective potential of *O. forsskalii* extract against ethanol.

## 1. Introduction

A peptic ulcer is a lesion in the gastrointestinal tract (GI) caused by the destruction of digestive enzymes, such as gastric acid and pepsin. This condition usually affects the upper part of the small intestine and the inner lining of the stomach and commonly occurs in the stomach, duodenum, and esophagus. Peptic ulcers affect approximately 4% of the world’s population [1]. The use of nonsteroidal anti-inflammatory drugs (NSAIDs), infection with *Helicobacter pylori* (*H. pylori*)*,* and cigarette smoking cause between 89% and 95% of severe upper gastrointestinal ulcers [2]. NSAID use is associated with duodenal and gastric ulcers and can lead to life-threatening complications of ulcers. *Helicobacter pylori* is a spiral-shaped, Gram-negative bacterium that is the most common cause of gastric ulcers in the first part of the small intestine, i.e., the stomach or duodenum [3].

Several synthetic over-the-counter peptic ulcers are available for treatment [4,5], but their efficacy is often exceeded by various side effects [6,7]. Therefore, an extensive search for peptic ulcer drugs from natural sources has been initiated to replace the drugs with uncertain safety and efficacy. Medicinal plants are safe, cheap, effective, and available sources of biologically active molecules, and their activity is mainly related to the presence of active secondary metabolites [8,9]. Various secondary metabolites have been investigated in numerous studies focusing on gastroprotective and antiulcerogenic properties [9,10]. Among the secondary metabolites, alkaloids [11], terpenes and terpenoids [12], flavonoids, phenolic acids, and tannins [13,14] are noteworthy. The antiulcerogenic effects of various medicinal plants have been demonstrated in animal studies using ethanol-, stress-, and indomethacin-induced ulcer models [15,16].

The Aizoaceae family is considered one of the most diverse and abundant families in the Arabian region. However, although several plants of this family are mentioned in the literature, they are still poorly studied for their medicinal value [17]. Some plant extracts (range 250–500 mg/mL) from the Azocea family were found to have antiulcer activity [18,19].

Bedouin tribes in northern Saudi Arabia mainly use seeds of *Opophytum forsskalii* (Hochst. ex Boiss.) N.E.Br (*O. forsskalii*; Synonym: *Mesembryanthemum cryptanthum* Hook.f., family: Aizoaceae) because they are rich in carbohydrates, proteins, and lipids. Moreover, these people mix the seed powder with dates and butter to prepare a traditional delicacy called pakilla [20]. In addition to the seeds, the aerial parts of the same or related species or other plants in the Aizoaceae family are also used as agents for hyperlipidemia, diuretics, antioxidants, cancer, rheumatism, laxatives, anti-inflammatories, and antimicrobials [21,22]. Abdel-Farid et al. reported that *O. forsskalii* has high antioxidant potential, followed by the seeds and fruits [20]. In Saudi Arabia, *O. forsskalii* grows abundantly in the northeastern part of the Aljouf region, especially in the Al-Adare and Bassita regions. The plant also occurs in other Arab regions such as Egypt, Qatar, Bahrain, and Palestine [23]. In addition, *O. forsskalii* has various medicinal properties such as antioxidants, cancer prevention, improvement of cholesterol and creatinine, antimicrobial activity, stomach pain, cytotoxic activities, etc., and has been widely used by Arabs [20,23,24].

Various analytical methods have been used to study secondary metabolite profiles [25,26]. Liquid chromatography-mass spectrometry (LC-MS) has high selectivity and sensitivity and allows the analysis of non-volatile, unstable, and high-molecular-weight compounds without derivatization. LC-MS is the most commonly used method for secondary metabolite profiling of plants [27]. Hamed et al. studied LC-MS analysis of 60% MeOH extract of Samh flour (*Mesembryanthemum forsskalei*) and identified 43 secondary metabolites, including several flavonoids and their glycosides [28]. However, according to our literature search, there are no data on the profile of secondary metabolites of the aerial parts of *O. forsskalii*.

This study analyzed the profiles of active secondary metabolites in *O. forsskalii.* We performed LC-MS to investigate this phenomenon. The results can be used to understand the role of this plant in pharmacological activities. Currently, there is limited information on the antiulcerative activity of this plant. This study investigated the in vivo antiulcerative activity of an 80% ethanol extract of the aerial parts of *O. forsskalii* using indomethacin, stress, and ethanol or other necrotizing agents that can be used for appropriate applications in the treatment of ulcers.

## 2. Results and Discussion

### 2.1. LC-MS Analysis of the Extracts

**Figure 1:**
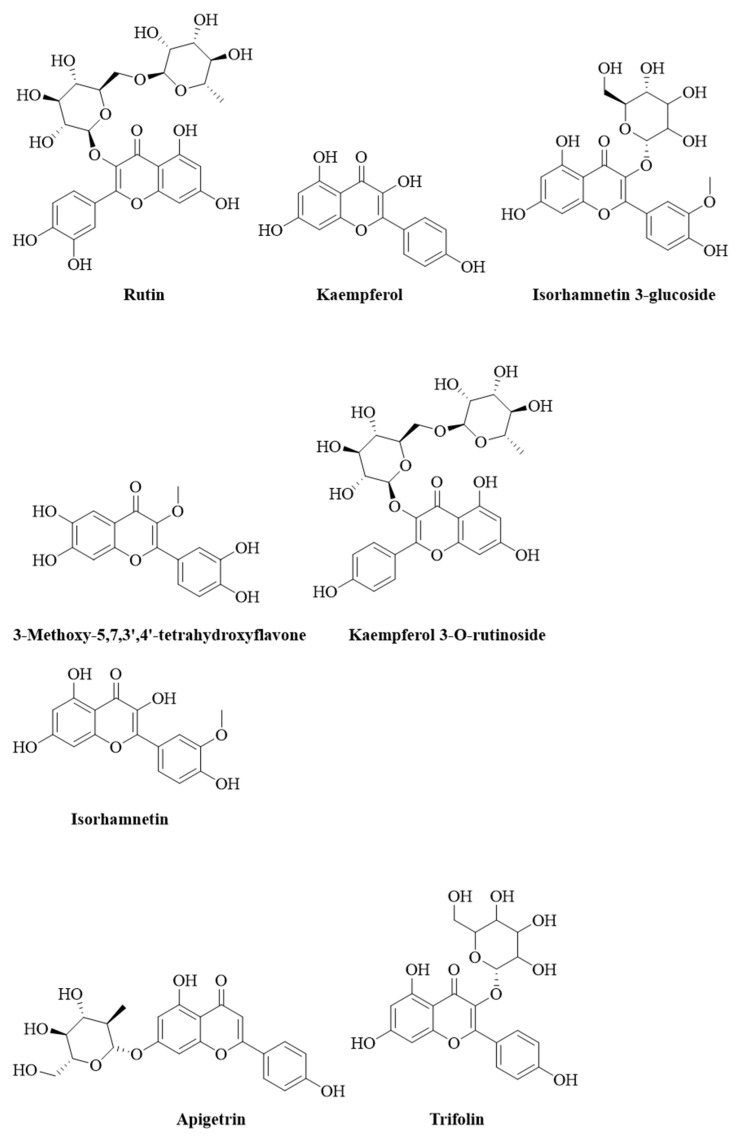

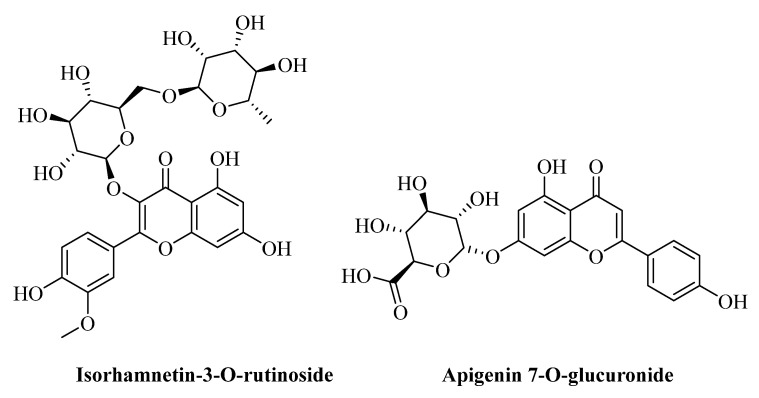
Chemical structure (drawn using PubChem Sketcher V2.4) of flavonoids and their glycosides.

Table 1 presents the secondary metabolites in the aerial parts of *O. forsskalii* and Figure 1 shows the structures of the flavonoids and its glycosides compounds.

Some phytoconstituents such as apigenin, camphorol, isorhamnetin, and rutin have been described in previous studies of *Mesembryanthemum forsskaolii* (syn. *O. forsskalii*) from Egypt [29,30]. Therefore, the metabolites reported in this species were compared in terms of molecular weight with the peaks reported in the same genus [17]. However, some of the metabolites of the extract could not be recognized because in-depth phytochemical studies involving column chromatography of pure compounds and structure elucidation by NMR analysis or other instrumental analyses are required.

It is well-documented that flavonoids and their glycosides are responsible for antioxidant, anticancer, and antiulcerogenic activity (Sharifi-Rad et al., 2018). Phytochemical screening of the alcoholic extract using LC-MS chromatogram analysis revealed the presence of about 30 compounds, most of which are flavonoids, glycosides, and fatty acids esters. The present study identified the most abundant class of secondary metabolites as flavonoids, their glycosides, and fatty acid ester. The flavonoids include rutin, apigetrin, kaempferol, tetrahydroxyflavone, trifolin, isorhamnetin, genistein, and their glycosides as kaempferol 3-O-rutinoside, isorhamnetin 3-glucoside, isorhamnetin-3-o-rutinoside, and apigenin 7-o-glucuronide were the most recorded secondary metabolites. The fatty acids esters such as (2Z)-3,7-dimethyl-2,6-octadien-1-yl 3-oxobutanoate, (8E)-2-amino-8-octadecene-1,3,4-triol, 2-(14,15-epoxyeicosatrienoyl) glycerol, 2-arachidonoyl glycerol, 27-norcholestane-3,7,12,24,25,26-hexol, ethyl linoleate, and 1-α-linolenoyl-2-arachidonoyl-sn-glycerol were identified in the alcoholic extract of *O. forsskalii*. Compounds such as coumarin, furoic acid esters, alkali metals and phthalate derivatives, sesquiterpene, steroids phenols, etc. were also found in the aerial parts of *O. forsskalii*. Ahmed et al. (2021) found that the active ingredients of antiulcer drugs can be attributed primarily to the presence of flavonoids [31]. Rutin, apigetrin, kaempferol, trifolin, isorhamnetin, apigenin, and genistein and its glycosides were the main flavonoid compounds used as active antiulcer agents [32,33]. The glycosides in addition to flavonoid compounds, fat acids [34], alkaloids [35], sesquiterpenes [36], steroids [37], and phenols [38] exhibit potent gastric ulcer or hepatoprotective properties. This study showed that the phytoconstituents were present in the aerial parts of *O. forsskalii*. These secondary metabolites could contribute to the antiulcer activity. However, the active metabolites in *O. forsskalii* did not show detailed pharmacological activities in the experimental animal studies, especially antiulcer activity. Therefore, the data indicated the presence of active metabolites that could support the use of *O. forsskalii* as an antiulcer agent. In the present study, several secondary metabolites were identified in the alcoholic extract of the leaves of *O. forsskalii* (Figure 1). Several studies reported that hymecromone, apigetrin, kaempferol, rutin, trifolin, isorhamnetin, apigenin, hexylresorcinol, genistein, etc. are nontoxic and have antiulcerogenic effects [39,40,41,42], which were also found in *O. forsskalii* [30]. *O. forsskalii* is used in traditional medicine, and in previous studies, the seeds of this plant [43] and several other plants of this family were found to be nontoxic [23,43,44]. In several studies, the major identified compounds flavonoids, their glyosides, and fatty acid esters have been reported to have gastroprotective potential against ethanol-induced ulcers, supported by the present study [45,46].

### 2.2. Antiulcerogenic Effect of O. forsskalii

The oral dose of indomethacin significantly damaged the glandular stomach of rats. The 400 mg/kg dose of *O. forsskalii* significantly reduced the development of gastric lesions in rat stomachs (28.33%); however, the 200 mg/kg dose of *O. forsskalii* had a limited preventive effect compared with the 400 mg/kg dose (Table 2).

Indomethacin, a non-selective cyclooxygenase inhibitor, caused gastric damage in rats by numerous methods, including reducing prostaglandin production, oversupplying leukotrienes, acting as a topical irritant, and reducing local blood flow [47]. In this model, rats pretreated with *O. forsskalii* provided significant protection. The gastroprotective effect of *O. forsskalii* extract could be attributed to increased gastric mucus, which produces prostaglandins and inhibits leukotrienes.

Table 3 shows that a dose of 400 mg/kg body weight of *O. forsskalii* extracts significantly low intraluminal hemorrhage value (1.66%) and ulcer development (18.33%) caused by hypothermic stress. At a 200 mg/kg dose, body weight, intraluminal bleeding, and UI were reduced, but the difference was not significant. Because of the repeatability of the data, ulcers caused by hypothermic stress were used as an experimental paradigm for evaluating antiulcer activity in rats [48]. Disruption of gastric mucosal microcirculation, acid secretion enhancement, and mucus production reduction is mediated by histamine release and abnormal gastric motility [49].

Free radicals may play an important role in stress-induced gastric injury [50]. Stress deactivates prostaglandin synthesis in the mucosa by accumulating hydrogen peroxide, an inhibitor of prostaglandin biosynthesis that generates reactive oxygen species (ROS) [51]. Moreover, a positive relationship between the amount of lipid peroxidation products in the gastric mucosa, a marker of oxidative stress, and gastric injury were demonstrated in rats subjected to cold stress [52]. The antioxidant activity of the extract of *O. forsskalii*, previously documented in *O. forsskalii* [20], and its anti-secretagogue effect could explain the protective effect of the animal against cold stress.

The effect of *O. forsskalii* extract on necrotizing agent-induced gastric lesions is presented in Table 4. Control rats were treated with 80% ethanol, 0.2 mol/L NaOH, and 25% NaCl, which resulted in severe gastric lesions in the glandular mucosa of the stomach. The UI was 6.83 ± 0.30, 5.83 ± 0.30, and 6.16 ± 0.30, respectively, in 80% ethanol, 0.2 mol/L NaOH, and 25% NaCl control rats 1 h after the necrotizing drugs were administered. Pre-treatment of rats with *O. forsskalii* extract at doses of 200 mg/kg of 80% ethanol, 0.2 mol/L NaOH, and 25% NaCl resulted in an UI of 5.33 ± 0.33 (*p* < 0.01), 5.33 ± 0.33, (*not significant*), and 5.00 ± 0.36 (*p* < 0.05), respectively. Similarly, pre-treatment of rats with *O. forsskalii* extract at doses of 400 mg/kg of 80% ethanol, 0.2 mol/L NaOH, and 25% NaCl revealed an UI 4.00 ± 0.36 (*p* < 0.001), 4.16 ± 0.30 (*p* < 0.01), and 4.33 ± 0.42 (*p* < 0.01), respectively, which inhibited the formation of gastric lesions. The transition from an erosive mucus layer to a gastric lesion is aided by gastric acid output. By contrast, proton pump inhibitors and histamine H2-receptor antagonists speed up the healing of gastric lesions or prevent mucosal injury [53].

*O. forsskalii* protected the gastric mucosa from ulcers caused by various necrotizing agents, such as ethanol and strong alkalis, in a statistically significant and dose-dependent manner. The use of ethanol-induced gastric ulcers to evaluate the gas-protective function is standard. When ethanol is metabolized in the body, free superoxide anions and hydroperoxyl radicals are produced. Free radicals generated by oxygen are involved in acute and chronic gastric ulcers [54]. Khazaei and Salehi pointed out that the development of ethanol-induced gastric lesions is multifactorial, with a decrease in gastric mucus and significant formation of free radicals leading to increased lipid peroxidation, which damages cells and cell membranes [55].

### 2.3. Assessment of the Oxidative Damage in Ethanol-Induced Ulcer

**Figure 2:**
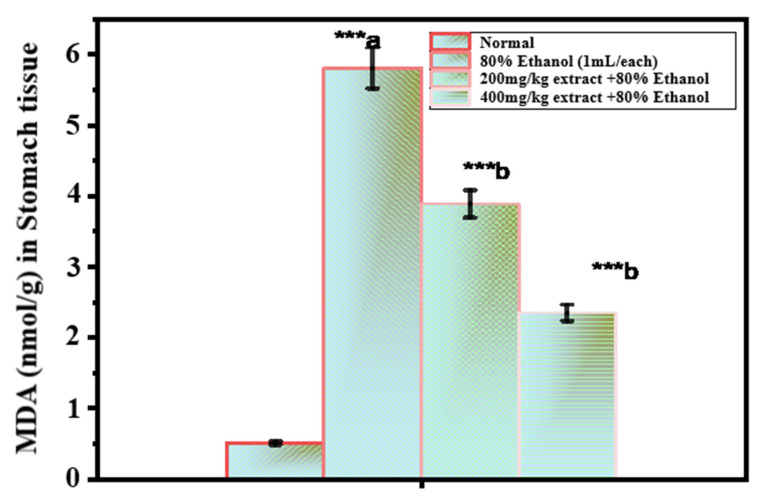
Effect of *O. forsskalii* extracts on the MDA concentration in gastric ulcer induced by 80% ethanol. Six rats were used in each group. *p* values (*** *p* < 0.001). Where, a: 80% ethanol treated group was statistically compared to the normal group; b: *O. forsskalii* treated groups were compared to the ethanol-treated group.

Figure 2 shows the MDA values in the gastric mucosa. The results used as the lipid peroxidation index were significantly higher in the ethanol-treated group than in the untreated control group (5.80 ± 0.34 μmol/g tissue; 0.54 ± 0.02 μmol/g tissue). At both doses (200 and 400 mg/kg), *O. forsskalii* (OF) significantly reduced the MDA content (3.88 ± 0.17 μmol/g and 2.34 ± 0.07 μmol/g, respectively). When the MDA content increases, free radicals, such as superoxide anion, hydrogen peroxide, and hydroxyl radicals, are formed. Cell degranulation is caused by the fact that these radicals increase the peroxidation of cell membrane lipids, leading to a loss of structural and functional integrity of cell membranes. When CAT does not scavenge hydrogen peroxide, it accumulates in the mitochondria and cytosol, increasing lipid peroxidation [56]. In addition, a clear relationship between the concentrations of lipid peroxidation end products in the gastric mucosa (a marker of oxidative stress) and gastric ulcers was found in stress-induced ulcers [49]. Since malondialdehyde (MDA) is the end product of lipid peroxidation, a decrease in the MDA concentration indicates a decrease in lipid peroxidation [57].

Lipid peroxidation was significantly inhibited by *O. forsskalii*. Moreover, *O. forsskalii* significantly suppressed MDA production from lipids reacting with thiobarbituric acid. Thus, the antioxidant property of *O. forsskalii* prevents the oxidative damage caused by alcohol intoxication. Moreover, *O. forsskalii* strengthens the mucosal barrier, the first line of defense against endogenous and foreign ulcerogenic chemicals, as evidenced by its antiulcerogenic activity [16].

**Figure 3:**
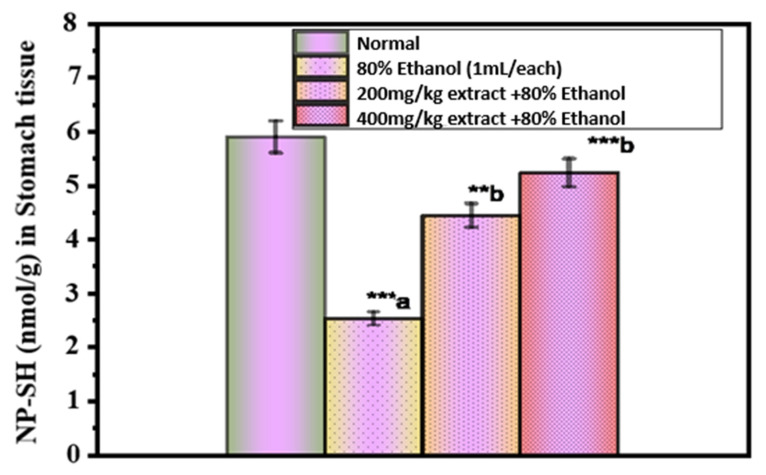
Effect of *O. forsskalii* extracts on the NP-SH concentration in gastric ulcer induced by 80% ethanol. Six rats were used in each group. *p* values (** *p* < 0.01, *** *p* < 0.001). Where, a: 80% ethanol treated group was statistically compared to the normal group; b: *O. forsskalii* treated groups were compared to the ethanol-treated group.

Figure 3 shows the concentration of NP-SH in the gastric mucosa. The concentration of NP-SH in the gastric mucosa of control rats was 5.80 ± 0.14 mmol/g tissue, which was significantly reduced to 2.53 ± 0.34 mmol/g (*p* < 0.001) after the administration of 80% ethanol. Ethanol-induced depletion of NP-SH was significantly increased in rats pretreated with an extract of *O. forsskalii* at both doses (200 and 400 mg/kg, respectively) (4.44 ± 0.44, *p* < 0.01; 5.23 ± 0.11, *p* < 0.001). Sulfhydryl compounds are critical for maintaining gastrointestinal integrity in living animals, especially when reactive oxygen species (ROS) play a role in causing tissue damage [58]. After ethanol administration, a sharp decrease in gastric NP-SH suggests a tremendous production of oxygen-generated free radicals.

The results of our studies support the findings of a previous study that again demonstrated sulfhydryl depletion in ethanol-induced gastric ulcers [59,60]. The reduction of glutathione exacerbates ulcerogenic-induced gastric mucosal injury in rats [61], whereas an increase in mucosal NP-SH leads to a gastroprotective effect. Our observations suggest that the extract of *O. forsskalii* protects the gastric mucosa.

**Figure 4:**
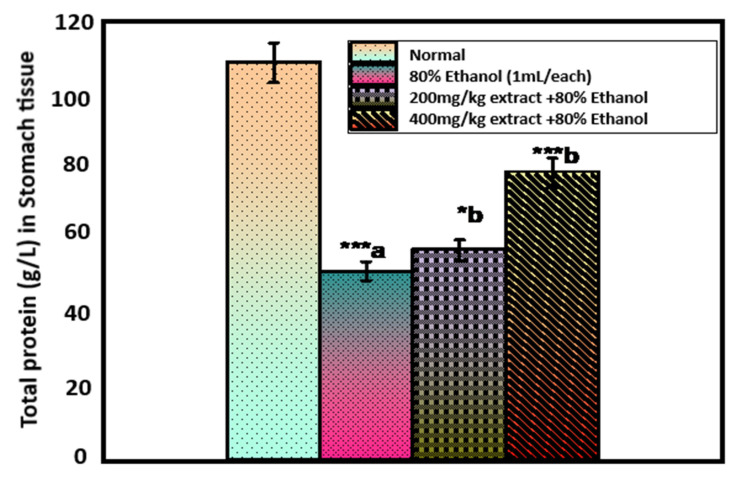
Effect of *O. forsskalii* extracts on the total phenol concentration in gastric ulcer induced by 80% ethanol. Six rats were used in each group. *p* values (* *p* < 0.05, *** *p* < 0.001). Where, a: 80% ethanol treated group was statistically compared to the normal group; b: *O. forsskalii* treated groups were compared to the ethanol-treated group.

Figure 4 shows the total protein (TP) level in gastric juice. The level of TP in the gastric juice of control rats was 108.57 ± 2.36 g/L gastric juice, which decreased significantly to 51.49 ± 1.82 g/L (*p* < 0.001) after administration of 80% ethanol. Pretreatment of rats with an extract of *O. forsskalii* at both doses (200 and 400 mg/kg) significantly increased the ethanol-induced decrease in TP (57.48 ± 1.38, *p* < 0.05; 78.63 ± 2.08 g/L, *p* < 0.001). Proteins play a key role in maintaining gastric integrity [62]. A significant decrease was observed in TP after ethanol administration, indicating malnutrition and disorders affecting the gastrointestinal system and interfering with the normal absorption of nutrients [63]. Our results are consistent with previous reports showing TP in ethanol-induced gastric lesions [64]. Treatment of rats with an extract of *O. forsskalii* resulted in a significant increase in TP. Our observations suggest that TP plays a role in protecting gastric mucosa by extracting *O. forsskalii*.

### 2.4. Histopathological Studies

**Figure 5:**
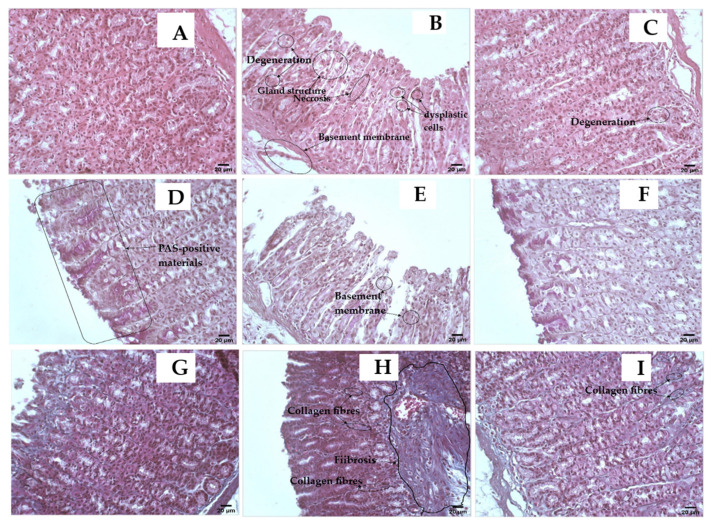
(**A**–**C**), Hematoxylin and eosin (H&E, magnification is ×400, scale bar is 20 µ) stain, where (**A**) is the standard stomach sample that displays a typical appearance of stomach tissue; (**B**) is an 80% ethanol stomach sample showing the toxic effects, which are indicated by loss of the ability for secretion and presence of vacuolation in the cytoplasm, necrosis, degeneration, dysplastic cells, basement membrane and atrophic mucosa detachment, and reduction in gland size or even absence of gland structure; (**C**) is 400 mg/kg *O. forsskalii* extract and 80% ethanol stomach sample showing considerable improvement and restoration of the secreting stomach microanatomical architecture. However, some glands suffer from the remaining effects of degeneration and atrophy of the stomach’s mucosa. (**D**–**F**) Periodic acid Schiff (PAS, magnification is ×400, scale bar is 20 µ) stain, where (**D**) is a normal stomach sample showing the high activity of mucosa in production. The presence of PAS-positive materials that exhibit a dark magenta color, especially towards the lumen, which is in the left half of this photomicrograph and basement membranes are intact for each gland; (**E**) is the 80% ethanol stomach sample showing a severe reduction and the absence of the mucosal activity of production of PAS-positive materials. Basement membranes also exhibit damage and weakness in multiple areas; (**F**) is 400 mg/kg *O. forsskalii* extract, and the 80% ethanol stomach sample exhibits considerable improvement in the inability of stomach gland cells to secrete PAS-positive materials, and the basement membrane also improved. (**G**–**I**) Mason trichrome (magnification is ×400, scale bar is 20 µ) stain, where (**G**) is a normal stomach sample showing a normal distribution of connective tissue; (**H**) is the 80% ethanol stomach sample: this group shows massive fibrosis due to the accumulation of collagen fibers in the submucosa and infiltration of collagen fibers into mucosa; (**I**) is 400 mg/kg *O. forsskalii* extract and 80% ethanol stomach sample, exhibiting considerable improvement and the presence of a few areas of collagen fiber infiltrates into the mucosa.

Pretreatment with the extract of *O. forsskalii* reduced ethanol-induced necrosis in the superficial layers of the gastric mucosa with congestion, as shown by the histopathological findings (Figure 5A–I). Specimens were prepared in 10% neutral buffered formalin and stained with hematoxylin and eosin (H&E), Masson’s trichrome, and periodic acid Schiff’s (PAS) for light microscopic studies [65]. Previous studies identified inflammation, hemorrhage, edema, and loss of epithelial cells in gastric tissue as features of ethanol-induced injury [66]. According to the results of this study, the extract of *O. forsskalii* significantly decreased the amount of infiltration of banded leukocytes caused by ethanol-induced gastric injury. Ethanol reduced the mucosal density at the mucosal margins, resulting in necrosis and ulceration, as suggested by Mahmood et al. [67]. PAS staining showed features of gastric regions where mucopolysaccharides are released, according to Tarnawski et al. [68].

Hematoxylin and eosin staining with ethanol showed cytoplasmic necrosis, degenerated cells, dysplastic cells, basement membrane, and a reduction in gland size [69]. The restriction of the microanatomical architecture using the alcoholic extract of *O. forsskalii* indicated the antiulcerogenic effect. PAS staining is used to detect mucopolysaccharides in gastric mucosa [68]. Due to the low PAS reactivity, the glycoprotein content of the gastric mucosa was lower in the II group. In contrast, the increased reactivity of PAS in the III group indicated an increase in glycoprotein. The Mason trichrome staining technique also revealed features of gastric sites where mucopolysaccharides are released. In individuals with ulcerative lesions in the stomach, gastrointestinal protective agents often increase PAS reactivity [70].

## 3. Materials and Methods

### 3.1. Materials

Sigma Aldrich (St.Louis, MO, USA) supplied the chemicals used in biochemical studies. The aerial parts of *O. forsskalii* were collected from Al-Kharj (the central region of Saudi Arabia). The sample was authenticated, and voucher number PSAU/PHARM/PF-101 was deposited at the Herbarium of the Pharmacognosy Department, Prince Sattam Bin Abdul-Aziz University (Al-Kharj, Saudi Arabia). The sample was dried in air and ground using a grinding machine (Geepas Countertop Mixer Grinder GSB5081, Dubai, United Arab Emirates) and passed to a mesh size of 40–60. The ethanol (70%) extracts were prepared by continuous shaking on an orbital shaker for 48 h each. A rotary evaporator (Buchi, Switzerland) was used to evaporate ethanol from the sample, and lyophilization using the freeze-drying (Freezone^®^ 2.5 model 76530, Labconco Corp., Kansas, MO, USA) technique was used to remove the water content. The final yield obtained was 14.8%. LC-MS was performed on a 1 mg/mL concentration of the extract. The extract was separately reconstituted in distilled water to prepare doses of 200 and 400 mg/kg body weight (b.w.) for the in vivo antiulcer activity.

### 3.2. Analysis of Secondary Metabolites Using LC-MS

**Figure 6:**
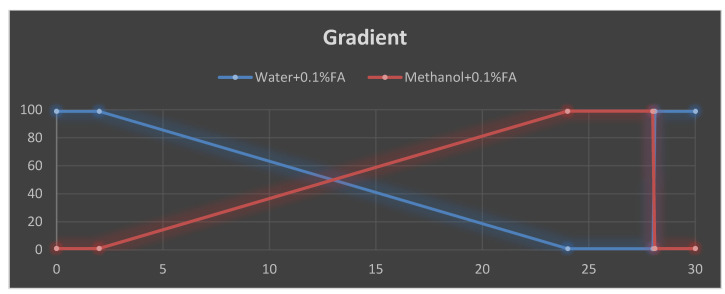
Gradient elution profile used for the LC-MS separation, decreasing order of mobile phase (water and 0.1% formic acid), increasing order of mobile phase (methanol and 0.1% formic acid).

According to a previous method, the secondary metabolites were investigated using LC-MS (Thermo Scientific, Waltham, MA, USA) instrumentation analyses [71]. The Orbitrap ID-X is a mass spectrometer that contains three mass analyzers and was used to analyze the m/z of the studied molecules. The Orbitrap IDX spectrometer could reach a high resolution (>120,000) and reliable mass accuracy (<3 ppm mass error). The mass spectrometer was calibrated using a purchasable ’Calibration Mix ESI (Thermo Scientific)’ by following the manufacturer’s guidelines Electrospray ionization in the positive mode (ESI+) was applied for the studied compounds. The following parameters were applied: mobile phase (Figure 6), vaporized temperature = 100 °C, voltage = 3500 V, sheath gas = 30, auxiliary gas: 15, ion source fragmentation = 35 V, capillary temperature = 300 °C. Here, 10 µL of the total extract was injected through a loop injection into a C18 column using an independent UHPLC pump.

UHPLC: The extract was automatically infused (10 µL) through the UHPLC system using a C18 column (Acquity CSH 100 × 2.1 mm, 1.7 µm) for the separation. The flow rate was set to 0.5 mL/min, and a gradient was applied for the separation as follows:

Data Processing: Compound discoverer 3.1 was used to treat and process the data using mzCloud Mass Spectral Library and local compound databases (exact mass or formula).

### 3.3. In Vivo Antiulcer Assay

#### 3.3.1. Animal Care

The instructions for the care and use of laboratory animals published by the College of Pharmacy (PSAU) were followed. Ethical clearance was obtained from the Department of Pharmacology, Prince Sattam bin Abdul-Aziz University (PSAU) and the standing committee of bioethics research (SCBR-009-2022) before the commencement of the research. Rats were procured, housed in plastic cages, and acclimatized for a week at the Animal Holdings of the Department of Pharmacology, PSAU, KSA, before research. The rats were kept at room temperature, fed a standard animal diet, and allowed free access to clean water. Sixty (60) albino Wistar rats (200 g) were obtained from the Animal House of the College of Pharmacy, PSAU. These animals were kept in clean, gauzed cages and acclimatized for two weeks at the animal house under standard temperature (25 ± 3 °C) and a 12:12 h light/dark periodicity. The animals were allowed free access to standard pellets and fresh water ad libitum. All the animals were handled in this study according to institutional guidelines describing the use of rats for studies.

#### 3.3.2. Indomethacin-Induced Gastric Lesion

An indomethacin-induced gastric lesion assay was used on the animals according to the procedure described by Alqasoumi et al., 2009 [16]. The rats were fasted for 36 h and categorized into 3 groups (*n* = 6), and orally given 30 mg/kg body weight of indomethacin suspended in 1.0% carboxymethylcellulose (CMC) in water (6 mg/mL); an equivalent amount of vehicle was used to treat control rats. The sample was given at 200 and 400 mg/kg doses, 30 min before indomethacin administration. Six hours following treatment, their stomachs were removed and checked for ulcers after cleaning with normal saline.

#### 3.3.3. Hypothermic-Restrained Stress-Induced Ulcers

A hypothermic-restrained stress-induced ulcer assay was used on the animals according to the method described by Alqasoumi et al., 2009 [16], with slight modifications. The animals were fasted for 36 h but given unlimited water. The rats were categorized into three groups (*n* = 6), restrained in restraint cages, and placed inside a vented refrigerator kept at 31 °C for 3 h, 30 min after oral administration of the extract (200 and 400 mg/kg). The stomachs of the animals were removed and assessed for ulceration and the severity of intraluminal hemorrhage using the arbitrary scale proposed by Chiu et al., in which 0 indicates no blood; 1 indicates that thin blood follows rugae; 2 indicates that thick blood follows the rugae; 3 indicates that thick blood follows the rugae with blood clots in specific regions; and 4 indicates that thick blood covers the whole gastric mucosal surface.

#### 3.3.4. Gastric Lesions Induced by Necrotizing Agents

A necrotizing agent, such as 80% ethanol, 0.2 mol/L NaOH, and 25% NaCl induced ulcer assays, was performed on the animals according to the method described by Al Mofleh et al. with slight modifications [63]. The rats were fasted for 36 h and then categorized into 9 groups (*n* = 6). Animals were categorized as follows: in groups 1, 2, and 3, the ulcer control was given saline only for the assessment of 80% ethanol, 0.2 mol/L NaOH, and 25% NaCl necrotizing agents, respectively; groups 4, 5, and 6 were treated with 200 mg/kg BW for the assessment of 0.2 mol/L NaOH and 25% NaCl necrotizing agents; whereas groups 7, 8, and 9 were treated with 400 mg/kg BW for the assessment of 0.2 mol/L NaOH and 25% NaCl necrotizing agents. All treatments were administered intragastrically for 8 days, and the gastric ulcers were created using a solution of 1 mL/animal of necrotizing agents 80% ethanol, 0.2 mol/L NaOH, and 2% NaCl on the last day. An hour after the administration of necrotizing agents, the rats were killed. Their stomachs were excised, filled with 2.5 mL of a 4% formaldehyde solution, and placed in a formaldehyde beaker. Their stomachs were opened over the more significant curvature and cleaned with a 0.9% saline solution to eliminate the blood clots after 10 min. Each stomach sample was subsequently placed on a slide. The UI of each rate was computed using the following formula:

UI = (total area of mucosal lesion (mm^2^) × 100)/(total mucosal area (mm^2^)

#### 3.3.5. Assessment of the Oxidative Damage in Ethanol-Induced Ulcer

After measuring the UI, the stomachs were washed with 0.9% (*w*/*v*) NaCl and used to determine various biochemical parameters.

NP-SH estimation was performed according to the previous method described by Sedlak and Lindsay [72]. Ice-cold 0.02 mmol/L ethylenediaminetetraacetic acid (EDTA) and the glandular portion of the stomach were homogenized in a Potter-Elvehjem type C homogenizer. Here, 5 mL of homogenate was combined with distilled water (4 mL) and 50% trichloroacetic acid (TCA, 1 mL). The tubes were centrifuged at 3000 r/min after being shaken intermittently for 10 min. Next, 2 mL of supernatant and 4 mL of 0.4 mol/L Tris buffer (pH 8.9) were mixed and agitated before the addition of 1 mL of 5,5’-dithio-bis (2-nitrobenzoic acid) (DTNB). The absorbance was measured at 412 nm against a reagent blank 5 min after DTNB was added.

Malondialdehyde (MDA) estimation was performed using the method described by Dursun et al. [73]. The stomach was removed and homogenized in 0.15 mol/L KCl (at 4 °C) to produce a 10% w/v homogenate. In a metabolic shaker, 1-mL aliquots of homogenate were incubated for 3 h at 37 °C. Next, 1 mL of 10% aqueous TCA was added and stirred. The mixture was then centrifuged for 10 min at 800 r/min and 1 mL of the supernatant was extracted and combined with 1 mL of water containing 0.67% thiobarbituric acid (TBA) for 10 min in a boiling water bath. After cooling, the liquid was diluted with 1 mL of distilled water. The absorbance was measured at 535 nm against a reagent blank. The MDA (nmol/g wet tissue) amount was estimated by referencing a standard curve of the MDA solution.

TP estimation was performed using the method described by Lowry et al. [74]. The alcoholic precipitate generated by mixing 90% alcohol with gastric juice in a 9:1 ratio was used to measure the dissolved proteins in gastric juice. The mixture of 1 mL of 0.1 N NaOH and 1 mL of alcoholic gastric juice was placed in a test tube. Subsequently, 0.05 mL was sampled, and 4 mL of the alkaline combination was added and allowed to stand. After 10 min, 0.4 mL of phenol reagent was added and allowed to rest for another 10 min for color development. The absorbance was measured at 610 nm against a reagent blank. The protein content was estimated by plotting a standard curve constructed with bovine albumin and expressed in g/L of gastric tissues.

#### 3.3.6. Histopathological Evaluation

Stomach tissue samples with a 3–5-cm thickness were obtained from the 3 animal groups and labeled carefully. Subsequently, these samples were immersed in a sufficient amount of 10% formalin solution. Tissues were prepared by the automatic tissue processing machine (ASP300s, Leica Biosystems, Buffalo Grove, IL, USA). The samples were then fixed in paraffin wax blocks using a rotary microtome. Following, 5-µm-thick sections were prepared (SHUR/Cut 4500, TBS, Sanford, NC, USA). The slide was stained by the hematoxylin and eosin technique described by Bancroft and Layton [75].

### 3.4. Statistical Analysis

The table and figure values are expressed as a mean and standard deviation. A one-way analysis of variance (ANOVA) was used to assess the data, followed by a student’s t-test. In the analysis, the 80% ethanol group *p*-values were compared with the normal, and test group *p*-values were compared with the 80% ethanol group, and * *p* < 0.05 was referred to as statically significant; ** *p* < 0.01 referred to very significant; and *** *p* < 0.001 as highly significant.

## 4. Conclusions

Extracts of *O. forsskalii* contain pharmacologically active metabolites such as flavonoids, alkaloids, steroids, coumarin, furoic acid, sesquiterpenes, and phenolic compounds. In animal models, these compounds exhibit potential pharmacological properties for preventing ulcers induced by indomethacin, stress, and the necrotizing agent ethanol. The extract is not toxic in normal cells and is not toxic in animal studies. Groups pretreated with extracts of *O. forsskalii* could inhibit MDA production and stimulate the secretion of NP-SH and TP. Moreover, this plant extract did not show any damage in the histopathological study, confirming that this plant contains potential active ingredients against gastric ulcers and can be used to discover and develop new drugs.

## Figures and Tables

**Table 1:** Active metabolites were obtained from *O. forsskalii* using LC-MS analysis.

No.	Name of Compound	RT	Proposed Formula	Theoretical Mwt.	M + H	Observed Mwt.	Mass Error (ppm)	Chemical Class
1	Nylon cyclic dimer	2.06	C_12_H_22_N_2_O_2_	226.1681	227.1754	226.1676	−2.3434	Macrocyclic lactam
2	4-Amino-3-[(1-carboxyvinyl)oxy]-1,5- cyclohexadiene-1-carboxylic acid	7.04	C_10_H_11_NO_5_	225.0638	226.0711	225.0633	−2.4438	Carboxylic acid derivative
3	Hymecromone	7.93	C_10_H_8_O_3_	176.0473	177.0546	176.0468	−2.9538	Coumarin
4	Rutin	9.48	C_27_H_30_O_16_	610.1537	611.1610	610.1532	−0.8522	Flavonoid
5	Hexyl 2-furoate	9.98	C_11_H_16_O_3_	196.1100	197.1173	196.1094	−2.9575	Furoic acid esters
6	(+)-Actinodaphnine	10.80	C_18_H_17_NO_4_	311.1158	312.1231	311.1153	−1.5750	Alkaloid
7	3-Methoxy-5,7,3’,4’-tetrahydroxyflavone	11.07	C_16_H_12_O_7_	316.0585	317.0658	316.0580	−1.6769	Flavonoid
8	Apigetrin	12.05	C_21_H_20_O_10_	432.1057	433.1130	432.1052	−1.1340	Flavonoid
9	Kaempferol	12.42	C_15_H_10_O_6_	286.0477	287.0550	286.0471	−1.9927	Flavonoid
10	kaempferol 3-*O*-rutinoside	12.42	C_27_H_30_O_15_	594.1587	595.1661	594.1583	−0.7574	Flavonoid
11	Trifolin	12.43	C_21_H_20_O_11_	448.1006	449.1077	448.0999	−1.5621	Flavonoid
12	Isorhamnetin 3-glucoside	12.50	C_22_H_22_O_12_	478.1111	479.1184	478.1106	−1.1504	Flavonoid
13	Isorhamnetin	12.51	C_16_H_12_O_7_	316.0585	317.0657	316.0579	−1.8984	Flavonoid
14	Isorhamnetin-3-*O*-rutinoside	12.52	C_28_H_32_O_16_	624.1690	625.1765	624.1687	−0.5127	Flavonoid
15	Apigenin 7-*O*-glucuronide	12.58	C_21_H_18_O_11_	446.0852	447.0925	446.0847	−1.1881	Flavonoid
16	Hexylresorcinol	14.69	C_12_H_18_O_2_	194.1307	195.1380	194.1301	−2.8847	Phenolic
17	Genistein	14.79	C_15_H_10_O_5_	270.0529	271.0602	270.0523	−2.1477	Flavonoid
18	Hexadecasphinganine	15.01	C_16_H_35_NO_2_	273.2668	274.2742	273.2663	−1.6833	Sphingoid
19	Botrydial	15.85	C_17_H_26_O_5_	310.1782	311.1854	310.1776	−1.9666	Sesquiterpene
20	Butylparaben	16.14	C_11_H_14_O_3_	194.0943	195.1016	194.0937	−2.9367	Benzoic acid derivative
21	(2Z)-3,7-Dimethyl-2,6-octadien-1-yl 3- oxobutanoate	17.11	C_14_H_22_O_3_	238.1569	239.1643	238.1565	−1.8475	Fatty acids ester
22	(8E)-2-Amino-8-octadecene-1,3,4-triol	17.61	C_18_H_37_NO_3_	315.2774	316.2846	315.2768	−1.8714	Fatty acids ester
23	Phytosphingosine	18.31	C_18_H_39_NO_3_	317.2930	318.3004	317.2926	−1.2922	Amino alcohol
24	2-(14,15-Epoxyeicosatrienoyl) glycerol	22.36	C_23_H_38_O_5_	376.2590	377.2663	376.2585	−1.4086	Fatty acids ester
25	2-Arachidonoyl glycerol	23.10	C_23_H_38_O_4_	378.2748	379.2820	378.2742	−1.5597	Fatty acids ester
26	27-Norcholestane-3,7,12,24,25,26-hexol	23.36	C_26_H_46_O_6_	454.3272	455.3345	454.3266	−1.2326	Fatty acids ester
27	Ethyl Linoleate	23.56	C_20_H_36_O_2_	308.2717	309.2790	308.2712	−1.7517	Fatty acids ester
28	Bis(2-ethylhexyl) phthalate	23.79	C_24_H_38_O_4_	390.2772	391.2845	390.2767	−1.3580	Phthalate derivative
29	1-α-Linolenoyl-2-arachidonoyl-sn-glycerol	26.33	C_41_H_66_O_5_	638.4891	639.4963	638.4885	−0.9241	Fatty acids ester
30	7alpha,25-Dihydroxycholesterol	26.82	C_27_H_46_O_3_	418.3447	419.3521	418.3443	−1.0757	Steroid

**Table 2:** Effect of *O. forsskalii* on indomethacin-induced gastric mucosal lesions.

Treatments	Dose mg/kg	Ulcer Index (Mean ± SE)
Control (Indomethacin Only)	30	37.33 ± 1.25
*O. forsskalii* Extract + Indomethacin	200	33.33 ± 0.49 *
*O. forsskalii* Extract + Indomethacin	400	28.33 ± 1.68 **

*p* values, * *p* < 0.05, ** *p* < 0.01, test groups were compared with the stress group.

**Table 3:** Effect of the *O. forsskalii* extract on hypothermic-restrained stress-induced intraluminal bleeding and gastric lesions in rats.

Treatments	Dose mg/kg	Intraluminal Bleeding Score Mean ± SE	Gastric Lesions Ulcer Index Mean ± SE
Control (stress only)	-	3.50 ± 0.42	25.20 ± 3.24
*O. forsskalii* extract + Stress	200	3.16 ± 0.30	23.50 ± 0.42
*O. forsskalii* extract + Stress	400	1.66 ± 0.21 **	18.33 ± 0.84 ***

*p* values, ** *p* < 0.01, *** *p* < 0.001, test groups were compared with the stress group.

**Table 4:** Effect of *O. forsskalii* on gastric lesions induced by necrotizing agents.

Treatments	Dose mg/kg	Ulcer Index (Mean ± SE)		
		80% EtOH	0.2 mol/L NaOH	25% NaCl
Control (80% Ethanol only)	1 mL/Rat	6.83 ± 0.30	5.83 ± 0.30	6.16 ± 0.30
*O. forsskalii* extract + 80% Ethanol	200	5.33 ± 0.33 **	5.33 ± 0.33	5.00 ± 0.36 *
*O. forsskalii* extract + 80% Ethanol	400	4.00 ± 0.36 ***	4.16 ± 0.30 **	4.33 ± 0.42 **

*p* values, * *p* < 0.05, ** *p* < 0.01, *** *p* < 0.001, test groups were compared with the necrotizing agent group.

## Data Availability

The data presented in this study are available in this article.

## Sample Availability

Samples of the plants are available from the authors.
